# Investigating the self-assembling of nicotinic hydrazide-based amphiphile into nano-range vesicles and its amphotericin B loading applications

**DOI:** 10.1080/10717544.2023.2174205

**Published:** 2023-02-13

**Authors:** Kashif Hussain, Abdul Jabbar, Khwaja Ali Hasan, Muneeb Ali, Zaheer Ul-Haq, Muhammad Raza Shah, Saeed Ahmad Khan, Md Abdur Rashid, Mohsin Kazi, Muhammad Naseer Abbas

**Affiliations:** a H.E.J. Research Institute of Chemistry, International Centre for Chemical and Biological Sciences, Karachi University, Karachi, Pakistan; b Molecular Biology & Structural Biochemistry Research Laboratory, Department of Biochemistry, University of Karachi, Karachi, Pakistan;; c Department of Pharmacy, Kohat University of Science and Technology, Kohat, Pakistan; d Division of Molecular Pharmaceutics and Drug Delivery, College of Pharmacy, The University of Texas at Austin, Austin, Texas, USA; e Pharmacy Discipline, Faculty of Health, School of Clinical Sciences, Queensland University of Technology, Brisbane, Queensland, Australia; f Department of Pharmaceutics, College of Pharmacy, King Khalid University, Guraiger, Abha, Saudi Arabia; g Department of Pharmaceutics, College of Pharmacy, King Saud University, Riyadh, Saudi Arabia; h Department of Pharmacy, Kohat University of Science and Technology, Kohat, Khyber Pakhtunkhwa, Pakistan

**Keywords:** Synthesis, CMC, self-assembly, biocompatibility, drug delivery

## Abstract

Most of the drugs are hydrophobic and have low water solubility, therefore posing issues in their absorption and bioavailability. Nonionic surfactants improve the solubility of hydrophobic drugs by entrapping them in their lipid bilayers. Two nonionic surfactants NODNH-16 and NODNH-18 are synthesized and characterized using different techniques i.e. EI-MS, ^1^H NMR, and FTIR. These newly synthesized surfactants were screened for blood hemolysis assay and cell toxicity studies using the NIH/3T3 cell line to assess their biocompatibility. Then amphotericin B was loaded into niosomal vesicles, and the drug entrapment efficiency of these surfactants was measured using UV–visible spectroscopy. The morphology of drug-loaded niosomes of synthesized surfactants was investigated using AFM, and their size, polydispersity, and zeta potential were measured with the Zetasizer instrument. Finally, a simulation study was performed to determine the pattern of self-assembly of the synthesized amphiphiles. Both synthesized nonionic surfactants showed good entrapment efficiency of 60.65 ± 2.12% and 68.45 ± 2.12%, respectively. It was also confirmed that both these synthesized nonionic surfactants were safe and biocompatible and showed less blood hemolysis (i.e. 21.13 ± 2.11% and 23.32 ± 2.45%) and higher 3T3 cells’ viability at 150 µg/mL concentration as compared to Tween®-80. The antifungal potential of amphotericin B-loaded niosomes has been evaluated against unicellular multi-fungal species, which showed a promising potential for fungicidal activity. These results are substantiated by constructing a safe vehicle system for drug delivery.

## 1. Introduction

Various drug delivery systems have been developed to overcome the harmful side effects, drug loss and degradation, and poor bioavailability issues associated with drugs (Torchilin, 2001; Panyam & Labhasetwar, 2003). Surfactants, soluble polymers, microcapsules, cell-penetrating peptides, micelles, lipoproteins, liposomes, and microparticles made up of biodegradable natural or synthetic polymers are examples of some of the most commonly used drug carriers (Reddy & Swarnalatha, 2010; Tagde et al., 2022). Each nanocarrier has its own merits and demerits therefore, the choice of selection of nanocarrier depends on relevant considerations (Torchilin, 2001). Colloidal particles such as niosome (Uchegbu & Vyas, 1998) and liposomes (Barratt, 2000) have substantial benefits over conventional drug delivery systems. Surface modification and composition of nanocarriers control the disintegration of drugs and affinity to target sites (Alsarra et al., 2005). Niosomes and liposomes are vesicular systems that can carry both hydrophobic and hydrophilic moieties by encapsulation and partitioning in their hydrophobic regions, respectively. Liposomes may be a unilamellar or multilamellar organoid structure made up of lipids (often phospholipids) assembled into a lipid bilayer (Francescangeli et al., 2003; Bansal et al., 2012). Liposomes have been extensively studied for their prospective applications in pharmaceuticals, such as increasing drug solubility, delivery, targeting, and modulating the release rates of medications; this is due to their ability to encapsulate a range of drugs (Sahoo & Labhasetwar, 2003; Peer et al., 2007). Despite remarkable advantages, their application in drug delivery is restricted mainly because of (i) hydrolytic or oxidative degradation in aqueous systems (Sagalowicz & Leser, 2010), (ii) sedimentation and aggregation during storage (Ulrich, 2002), (iii) and sterilization, large-scale production with adequate physicochemical stability issues (C. Hu & Rhodes, 1999). One alternative to liposomes is the formation of liposomes-like vesicles (Niosomes) from the mixture of nonionic surfactants (e.g. monoalkyl polyoxymethylene and dialkylpolyoxyethylene ethers) and cholesterol (Sahin, 2007; Varun et al., 2012; Abdelkader et al., 2014). From a technical point of view, niosome are promising nanocarriers and the best alternative to liposomes as they have significant stability and lack many of the drawbacks associated with liposomes (e.g. purity problem of phospholipids and high cost, less stability) (Alsarra, 2009; Wagh & Deshmukh, 2010). Furthermore, routine and large-scale production of niosome requires simple methods without the use of any harmful solvents as well as economically cost-effective (Meng & Russel, 2005). Amphotericin B is a polyene antibiotic having fungicidal potential and is also used as a gold standard to treat leishmaniasis (Lemke et al., 2010; Nasr et al., 2012; Van de Ven et al., 2012). However, amphotericin B administration causes toxicity to healthy cells and has poor aqueous solubility, so it is exclusively administrated intravenously (Van de Ven et al., 2012; Butani et al., 2014). As amphotericin B has great importance in treating endemic diseases in less developed countries, its oral dosage form is highly desirable because of its simplicity and accessibility (Ensign et al., 2012; Wang & Zhang, 2012). Here, we have synthesized two new nicotinoyl hydrazine-based amphiphilic nonionic surfactants having a lipophilic long tail of 16 carbons and 18 carbon chains. The pattern of self-assembly of these newly synthesized surfactants was assessed with a molecular dynamics simulation study. The hydrophobic drug amphotericin B is used as a model drug to determine the entrapment efficiency of these nonionic surfactants.

## 2. Methods and materials

### 2.1. Materials

In this research work, HPLC-grade solvents were used. 4 Hydroxybenzaldehyde, nicotinic hydrazide, Dulbecco’s modified Eagle’s medium (DMEM), K_2_CO_3_, polylysine (PLL), 3-(4,5-Dimethylthiazol-2-yl)-2,5-Diphenyltetrazolium Bromide (MTT), fetal bovine serum (FBS), 1-bromooctadecane, and 1-bromohexadecane were obtained from Sigma–Aldrich, Germany. Cholesterol and Tween®-80 were acquired from BDH, UK, and Merck, Germany through local suppliers. Sabouraud dextrose agar and Capek’s dox agar (Merck, Germany), standard fungicidal drugs including miconazole (GSK, Pakistan), nystatin (Wyeth, Pakistan), and amphotericin B (Am-B) (Abbott, Pakistan) were acquired from their vendors.

### 2.2. Synthesis of nonionic surfactants

**Figure 1.**
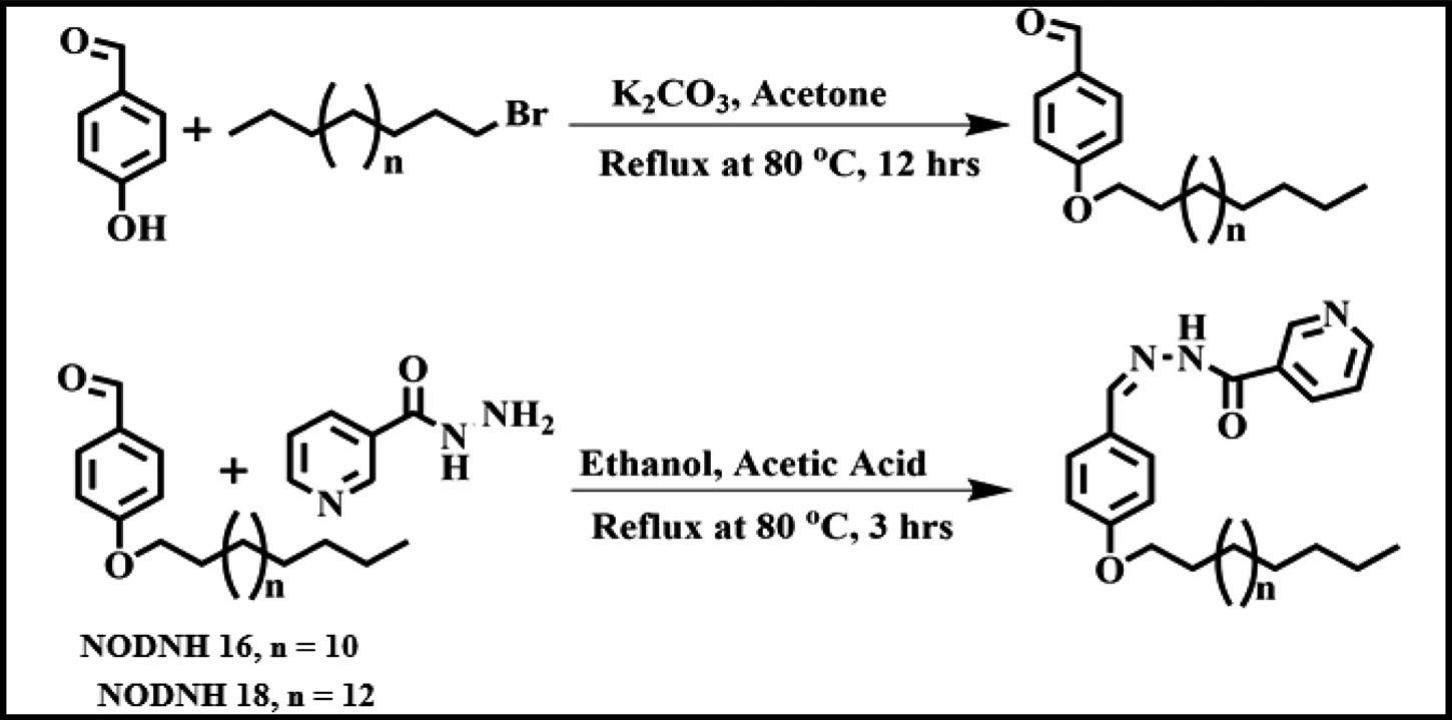
Synthesis of nonionic surfactants NODNH-16 and NODNH-18.

In the initial reaction, 122 mg (1.0 mmol) of 4-hydroxy benzaldehyde was reacted with 499.9 mg (1.5 mmol, 1.5 mol equivalent) of 1-bromohexadecane in 10 mL acetone in the presence of 345.5 mg (2.5 mmol, 2.5 mol equivalent) of K_2_CO_3_ and refluxed for 12 h. When the reaction was completed, it was cooled to ambient temperature, concentrated, and products were purified through column chromatography. Then, in a round-bottom flask (50 mL), 70 mg of nicotine hydrazide (0.5 mmol, 1 mol equivalent) and 187.2 mg of 4-(octadecyloxy) benzaldehyde (0.5 mmol) were added in 20 mL ethanol and stirred for 20 min at 80*°*C then added a few drops of anhydrous acetic acid and the reaction was refluxed for 3 h at the same temperature. The reaction was stopped, cooled down to ambient temperature, and filtered. The white powder of desired product (NODNH-18) was obtained with a 90% yield. In another reaction, the nonionic surfactant NODNH-16 was synthesized by following the same procedure. The white powder of desired product (NODNH-16) was obtained with an 84% yield, as shown in Figure 1.

**Yield:** for NODNH-16, 84%, m.p. 117.2–119.5 °C and for NODNH-18, yield: 90%, m.p. 118.5–120.8 °C.

### 2.3. Critical micellar concentration determination

The critical micellar concentration (CMC) of synthesized nonionic surfactants was determined using a UV–visible spectrophotometer (Shimadzu, UV-240). Solutions of various concentrations of the synthesized nonionic surfactants were prepared in ethanol ranging from 0.050 mM to 0.001 mM and then scanned in the UV–visible region (200 to 800 nm) by spectrophotometer. Absorbance at *λ*_max_ was plotted against the respective concentration of nonionic surfactants. Lines were drawn to connect respective concentrations against their absorption and CMC was assigned to the point where discontinuity occurs in the graph’s straight line (Ullah et al., 2015).

### 2.4. Surfactant vesicles

#### 2.4.1. Preparation of surfactant vesicles and drug loading

The drug-loaded vesicles of synthesized nonionic surfactants NODNH-18 and NODNH-16 were prepared through the hydration of the surfactant thin film method (Ali, Rehman et al., 2018; Ali, Shah et al., 2018). Briefly, cholesterol (7 mg), surfactant NODNH-18 or NODNH-16 (15 mg), and drug (amphotericin B; 5 mg) were made soluble in a binary solvent mixture of chloroform and methanol (7 mL:4 mL). The solvent was then evaporated through a rotary evaporator under reduced pressure to form a thin lipid layer film which was hydrated with 5 mL of deionized water. The size of vesicles was reduced using an ultrasonicator (LABSONIC L, USA) for 4 min at 25 °C. The drug-loaded vesicular suspension was saved for further experiments.

#### 2.4.2. Determination of morphology, size, polydispersity index, and zeta potential

The shape of drug-loaded niosomal vesicles of NODNH-18 and NODNH-16 was determined by atomic force microscopy, AFM (Agilent, 5500, USA) as reported (Imran et al., 2016). Briefly, a diluted drop of drug-loaded vesicular suspension of NODNH-18 and NODNH-16 was dropped on a mica slide and carefully dried in dust-free environment. The dried mica slide was placed under the microscope and read carefully in a non-contact mood. The mean size, hydrodynamic diameter, and zeta potential of drug-loaded vesicular suspension of both surfactants were measured by Zetasizer (Malvern Instruments, UK) according to previously reported methods (Hussain et al., 2022).

#### 2.4.3. Drug entrapment efficiency determination

The drug entrapment efficiency of synthesized nonionic surfactants NODNH-18 and NODNH-16 were assessed using the UV–visible spectrophotometric method. Briefly, 1 mL suspension of vesicles having 1 mg drug (amphotericin B) was taken in an Eppendorf tube and centrifuged for 15 min at 12,000 rpm using a centrifuge (Universal 16, Hettich, Germany). The drug-loaded vesicles settled to the bottom of the Eppendorf and formed a solid pellet. The procedure was repeated three times to completely remove all of the free drugs from the supernatant (Ali et al., 2017). The pellet was redispersed in methanol and read spectrophotometrically at 405 nm for amphotericin B quantification. The entrapment efficiency was measured using the following formula:

(1) $$\text{EE}\left(\%\right)=\frac{\text{Totla drug added}-\text{free drug}}{\text{Total drug used in the formulation}}\times 100$$

#### 2.4.4. In vitro drug release study

A drug release study from NODNH-16 and NODNH-18 vesicles was performed using a dialysis membrane. Five milliliters of amphotericin B-loaded vesicle suspension (1 mg/mL) was taken in a dialysis membrane (12,000 kDa pore size). The membrane was then dipped in a beaker having 50 mL buffer solution (PBS) of respective pH followed by shaking at 50 rpm/min at 37 °C. Then at specific times, aliquots of 2 mL were withdrawn and refilled with fresh buffer solution to prevent drug saturation in the media. The amount of drug release in each dissolution media was determined spectrophotometrically at 405 nm.

#### 2.4.5. Drug–excipients interaction study

Drug–excipients interactions in the formulation were assessed using FTIR spectroscopy (IR-470, Shimadzu, Kyoto, Japan). IR spectra of dried vesicles containing drugs and individual components of the vesicle, i.e. drug, cholesterol, and surfactants NODNH-18 and NODNH-16 were recorded from 400 to 4000 cm^−1^. Samples were prepared and analyzed with the KBr-disk method where KBr powder was used for the preparation of disks.

#### 2.4.6. Molecular dynamics simulation

To evaluate the self-assembling process and to investigate how the micelles’ shape depends on the molecular size of the synthesized surfactants, atomistic molecular dynamics (MD) simulation was used. All MD simulations were performed with GROMACS 2016.4 (Abraham et al., 2015) using a GPU system where every atom was explicitly represented. The OPLS_AA (Jorgensen et al., 1996), an all-atom force field for organic molecules was used to describe the bonded, non-bonded (Lennard–Jones; LJ), and Columbic interactions in a system. The extended simple point charge (SPC/E) water model was used to describe the solvent surrounding the micelles’ molecules (Berendsen et al., 1987). The molecular topologies were generated by the LigParGen server (Dodda et al., 2017). Fifty molecules of synthesized surfactant molecules were randomly inserted into a 10 nm^3^ cubic simulation box and subsequently solvated by the addition of discrete numbers of water molecules. All MD simulations were started with the random distribution of the surfactant molecules in the cubic periodic box which was produced using Gromacs utility ‘genbox’. Energy minimization was done for each molecule as a first step employing the steepest descent algorithm till the maximum force of the system came to <0.001 N/mol. After energy minimization, a constant number of particles (*N*), volume (*V*), and temperature (*T*) and the NPT ensemble (*N* = constant, *P* = pressure, and *T* = temperature) were sequentially carried out for 100 picoseconds each to equilibrate the system at 1 bar pressure and 298 K temperature. Micelles were formed at 30 ns and the period of the last 5 ns of this run was used for analysis. The inspection and visualization of trajectories and arrangement of amphiphiles in bulk water were performed with visual molecular dynamics VMD 1.9.1 (Humphrey et al., 1996).

### 2.5. Biocompatibility study

#### 2.5.1. Blood hemolysis assay

The newly synthesized nonionic surfactants NODNH-18 and NODNH-16 were screened for hemolysis assay against human blood. The fresh human blood for this study was obtained from the Center for Bioequivalence Studies and Clinical Research (CBSCR), University of Karachi. To separate red cells from the blood, it was centrifuged for 10 min at 700 *g*, and the supernatant was discarded. The separated RBCs were washed three times with phosphate-buffered saline (PBS; pH 7.4) and centrifuged in similar conditions. RBCs were dispersed in PBS in a 1:10 ratio (RBCs: PBS, wt/vol) and 4 mL of different concentrations of synthesized surfactants (62.5–1000 mg/mL) and 200 µL of RBCs were mixed. For reference standards, the commercially available Tween®-80 was screened. Samples were incubated at 37 °C for 4 h, then centrifuged to separate hemolyzed blood in the form of supernatant and read at 540 nm to determine free hemoglobin spectrophotometrically. The RBCs suspension was similarly mixed with water and PBS to obtain the 100% and 0% hemoglobin release. The following equation was then used to determine the % hemolysis:

(2) $$\text{Hemolysis}\;\left(\%\right)=\frac{\text{Abt}-\text{Abo}}{\text{Ab}100-\text{Abo}}\times 100$$

Abt, Ab0, and Ab100 indicate the absorbance of the test sample, PBS, and water, respectively (Cenni et al., 2008).

#### 2.5.2. Cell toxicity

To assess the cell line toxicity potential of synthesized nonionic surfactants, NIH/3T3 (8.0 × 10^3^) cells were rooted in 96-well plates containing DMEM (Dulbecco’s modified Eagle medium) supplemented with penicillin and streptomycin antibiotics (50 IU/mL each). Cells were incubated in cultured media for 24 h at 37 °C in a 5% CO_2_ humidified environment. After 24 h, cells were exposed to the same volume of fresh media containing synthesized surfactants NODNH-16 or NODNH-18 in 62.5–1000 μg/mL concentrations. Cells viability was assessed via the well-known 3-(4,5-dimethylthiazol-2-yl)c-2,5-diphenyltetrazolium bromide (MTT; Sigma, USA) assay. In short, 20 µL of MTT solution (0.5 mg/mL) was added to each well and incubated for 2 h in the same environmental condition as described for cell culturing. After incubation, the supernatant was removed from each well and 50 µL DMSO was added to each well. The formazan amount formed was determined using microplate readers (ELx808, BioTek, USA) at 670 nm. Untreated cells were taken as 100% viable and Tween®-80 was used as the reference standard. Percent cell viability for each sample was calculated using the following formula:

$$\text{Cells}\prime \text{viability}\;\left(\%\right)=\frac{\text{absorbance of sample}}{\text{absorbance of negative control}}\times 100$$

### 2.6. Biological activities

#### 2.6.1. Fungal strains, standard anti-fungal drugs, and amphotericin B vesicle

Human pathogenic fungal cultures i.e., *Candida albicans* ATCC 10231 and *Candida galeberata* ATCC 20001 obtained from Oxoid, UK, and grown onto sabouraud dextrose agar (SDA) (Merck, Germany) at 28 °C for 48 h. Multi-cellular and dimorphic plant-associated fungal isolates i.e. *Trichoderma longibrachiatum* **(**MW564026), *Purpureocillium lilacinum* (MW566158), *Aspergillus terreus* (MW570851), *Aspergillus flavus*, *Paecilomyces variotii*, and *Fusarium oxysporum*, and two yeast species *Meyerozyma guilliermondi* (MW564205) and *Saccharomyces cerevisiae* were grown onto sabouraud dextrose and czapek’s dox agar (Merck, Germany) for 2–7 days at room temperature (Colozza et al., 2012; Elefanti et al., 2013). Fungal strains were identified at the molecular level by the ITS gene sequencing and were submitted to the gene bank providing the accession numbers.

#### 2.6.2. Antifungal assay

The antifungal assay was performed using broad-spectrum fungicidal drugs and niosomal vesicles i.e. NODNH-16 and NODNH-18 containing encapsulated amphotericin B. Standard fungicidal drugs including miconazole (GSK, Pakistan), nystatin (Wyeth, Pakistan), and amphotericin B (Abbott, Pakistan) were reconstituted at a concentration of 1500 µg/mL for the assay (Magaldi et al., 2004; Colozza et al., 2012; Shah et al., 2021). Briefly, overnight cultures of *C. albicans* ATCC 10231, *C. galeberata* ATCC 20001, *M. guilliermondi* (MW564205), and *S. cerevisiae* grown in Sabouraud dextrose broth (Oxoid, U.K) and cells were harvested after centrifugation at 9000 *g*. Cell suspension equivalent to 0.5 McFarland standard (1.5 × 10^8^ CFU/mL), was prepared in 10 mL sterile phosphate buffer saline solution (PBS). Confluent lawns were prepared onto SDA plates and allowed to dry for 5–10 min. Standard fungicide at a final concentration of 15 µg and the vesicle formulations NODNH-16 and NODNH-18, comprising 60.89% (i.e. 9.13 µg) and 68.63% (i.e. 10.29 µg) encapsulated amphotericin B respectively, was dispensed into the wells (6 mm), bored with help of a sterile cork-borer, and plates were incubated at 28 °C for 24–48 h (Magaldi et al., 2004; Mady et al., 2018). For multi-cellular fungi, spore suspension (5 × 10^5^ spores/mL) was prepared in PBS from five days old cultures. Spores were inoculated with the help of a sterile swab on SDA and incubated at 25 °C for five to seven days. The antifungal activities were performed in triplicates. The results were interpreted and compared with the zones of inhibition in mm(±SD)produced by carrier vesicles and standard drugs as per CLSI (2012) guidelines (Mady et al., 2018).

#### 2.6.3. Antifungal index

The antifungal index (AFI) was calculated by the following formula.

AFI = Zone of inhibition of amphotericin B loaded liposomal vesicles/Zone of inhibition of amphotericin B. AFI was calculated in replicate experiments.

AFI values >1 indicate the niosomal vesicles are more active against the tested fungal species than the standard antifungal used.

### 2.7. Statistical analysis

All experiments were performed in triplicate and results were expressed as mean ± SEM. One-way analysis of variance (ANOVA) was used for statistical analysis using Graph Pad Prism software (Version 5). Less than or equal to .05 *p* values were considered statistically significant.

## 3. Results and discussions

### 3.1. CMC determination

**Figure 2.**
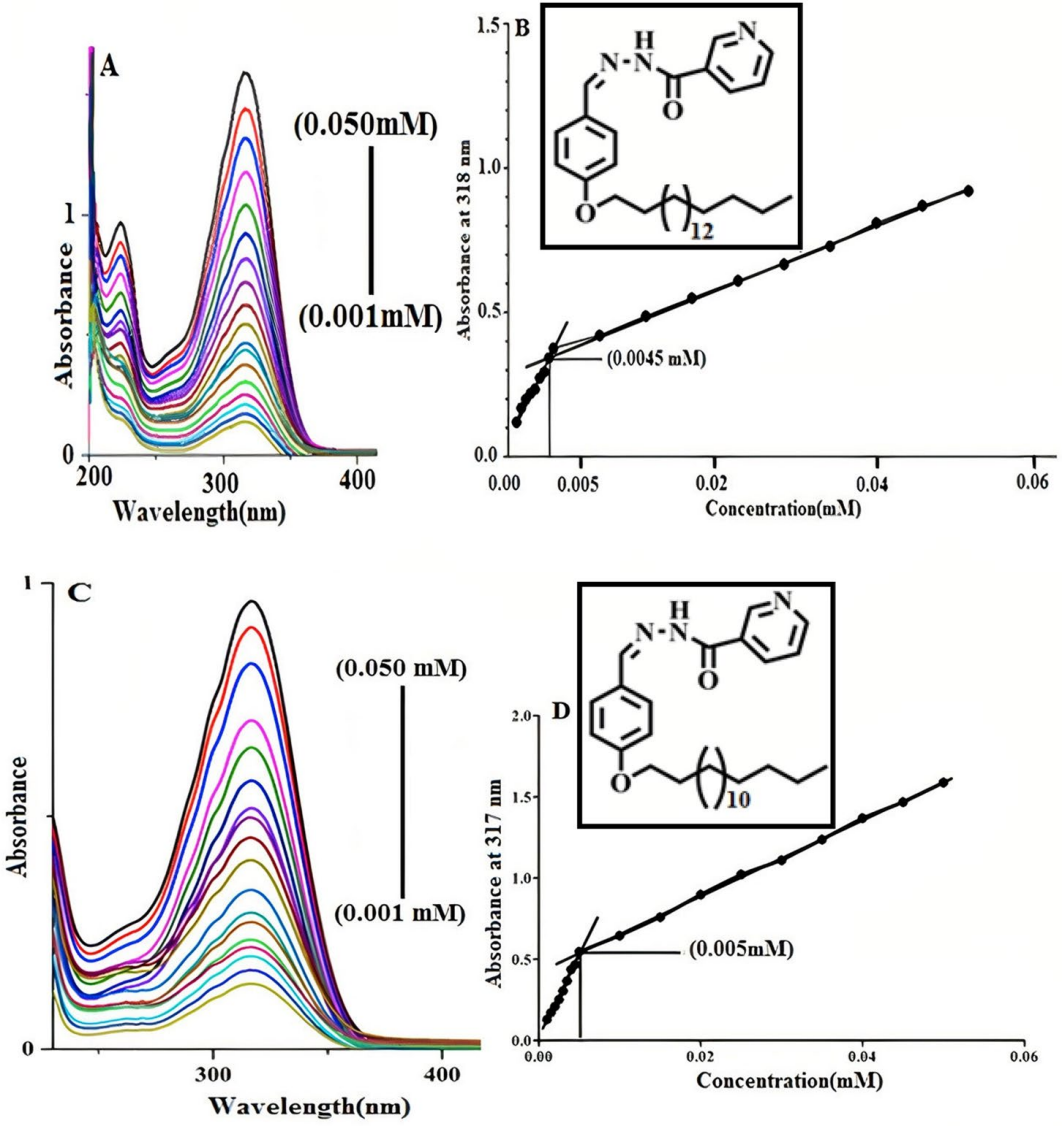
The CMC of synthesized nonionic surfactants NODNH-16 and NODNH-18.

The CMC of an amphiphilic molecule is the concentration above which the individual monomers of the surfactant or amphiphile start to assemble in micelles thus playing a vital role in the formation of micelles. A balanced hydrophobic and hydrophilic segment of amphiphilic molecules ensures the core/shell structured micelles formation in an aqueous medium. The aggregation behavior of nonionic surfactants is a function of the molecular weight of hydrophilic and hydrophobic segments (Griffin, 1949; Helenius & Simons, 1975). The determination of CMC by spectrophotometry is a simple and durable procedure. The absorbance cutoff is significant at the concentration at which micelles begin to form (Ali et al., 2017). This discontinuity in absorbance versus concentration can be used for the determination of CMC. For the determination of CMC of nonionic surfactants, different concentration solution of newly synthesize surfactants was prepared in ethanol ranging from 0.050 mM to 0.001 mM and run-on UV–visible spectrophotometer. The CMC of both synthesized amphiphilic molecules was determined by drawing a graph of absorbance against concentration. The amphiphilic molecule NODNH-18 showed absorbance max at 318 nm wavelength as shown in ***Figure 2***(A) and the value of CMC was measured as 0.0045 mM as depicted in Figure 2(B). Similarly, the NODNH-16 showed maximum absorbance at 317 nm wavelength and the CMC value was noted at 0.050 mM as shown in Figure 2(C,D). The chain length of the lipophilic carbon chain, the area of the hydrophilic head, and the volume of the hydrophilic head are some of the factors that affect the CMC of an amphiphile. In water, the CMC value for a surfactant increases with the effective size of the head groups and decreases with the molecular weight of the tail (Dan, 2017). The greater value of CMC of NODNH-18 was due to a larger chain length as reported elsewhere (Ali et al., 2017).

### 3.2. Surfactant vesicles

#### 3.2.1. Size, morphology, polydispersity, and zeta potential of drug-loaded vesicles

**Figure 3.**
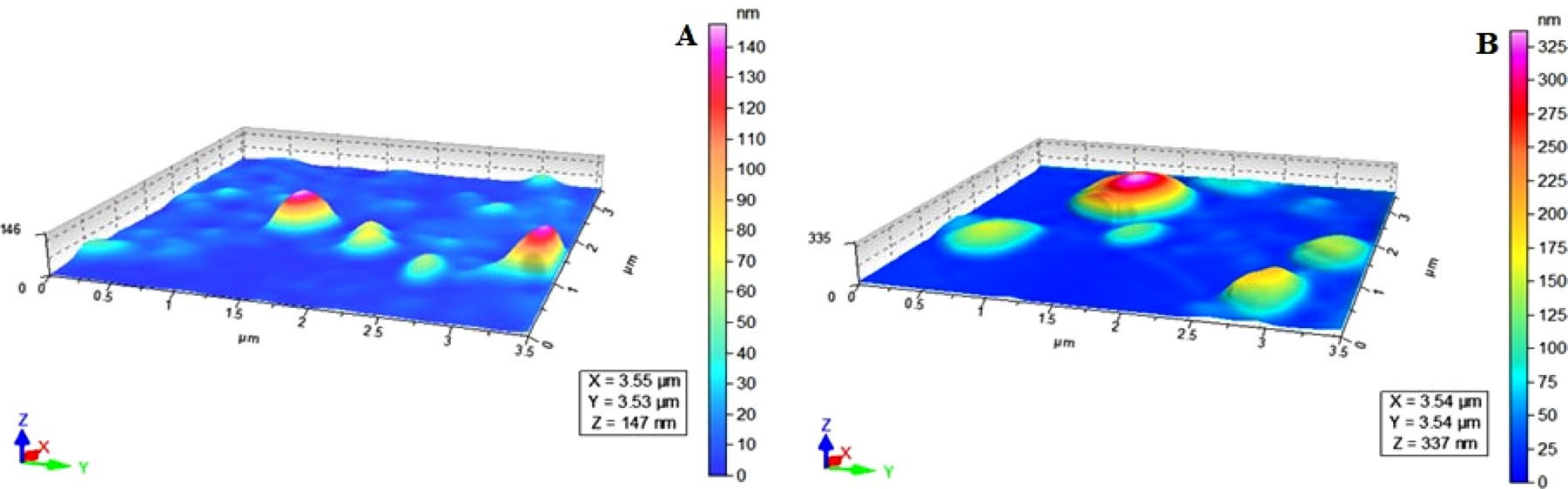
AFM images of niosomal suspension of NODNH-16 (A) and NODNH-18 (B).

The morphology of drug-loaded vesicles of newly synthesized nonionic surfactants NODNH-16 and NODNH-18 was assessed using atomic force microscopy (AFM). The drug-loaded vesicles of synthesized surfactants were found spherical as shown in ***Figure 3***. The size, polydispersity index, and zeta potential of drugs-loaded vesicles of synthesized surfactants NODNH-16 and NODNH-18 were measured with Zetasizer using the DLS technique. The average size of drug-loaded vesicles of NODNH-16 was 95.34 ± 2.21 nm and NODNH-18 was found to be 96.13 ± 2.35 nm as shown in ***Table 1*** (Hussain et al., 2022) The zeta potential and polydispersity index of NODNH-16-based drug-loaded vesicles were noted as –19.6 ± 0.9 mV and 0.28 whereas for drug-loaded vesicles of NODNH-18-based surfactant; the zeta potential and polydispersity index were found –18.8 ± 0.8 mV and 0.29 as shown in Table 1. The smaller average size of drug-loaded vesicles of newly synthesized nonionic surfactants NODNH-16 and NODNH-18 is of greater importance as the smaller-sized nanocarriers are reported to have longer blood half-lives. The Zeta potential of a system indicates the overall charge and stability of the nanosystem in liquids and more positive or more negative values of zeta potential are desired for its stability (Gaikwad et al., 2019). The size and zeta potential values of drug-loaded vesicles of the newly synthesized surfactants show that they form stable vesicles over a long period of storage. The stability of drug-loaded vesicles NODNH-16 and NODNH-18 was assessed over a month under storage conditions at 4 °C by monitoring the size, polydispersity, and zeta-potential. The drug-loaded vesicles presented a marvelous profile and didn’t find major changes in size, and PDI, as well with no considerable modification in surface charge. The good stability is most possibly due to negative zeta-potential which leads to greater stability (Ceren Ertekin et al., 2015; Khan et al., 2015; Rasul et al., 2020). Cholesterol was another significant component that influenced their physicochemical properties and enhanced the stability of niosomes (Rasul et al., 2020).

**Table 1.** Composition, average size, zeta potential, polydispersity index, and percent drug entrapment efficiency.

Sample	% Composition (Compound:Cholesterol:Drug)	Average size (nm)	Zeta potential	PDI	%Drug loading efficiency
NODNH-16	15:7:5	95.34 ± 2.21	–19.6 ± 0.9	0.28	60.89 ± 2.88
NODNH-18	15:7:5	96.13 ± 2.35	–18.8 ± 0.8	0.29	68.63 ± 3.43

#### 3.2.2. Drug entrapment efficiency

For proper characterization of a nano-drug delivery system, its capability of entrapping drug amounts is very important. The amphotericin-B entrapment efficiency by NODNH-16 and NODNH-18-based vesicles was determined following the separation of the un-entrapped drug by cold centrifugation. The entrapped drug in the sedimented vesicular pellet was determined by dissolving it in methanol and subsequent reading on UV-spectroscopy at a maximum wavelength of 405 nm. The amount of drug entrapped in newly synthesized surfactant NODNH-18 was 68.63 ± 3.43% and similarly, the drug entrapment efficiency of NODNH-16-based vesicles was 60.89 ± 2.88% (Table 1). Among other factors, the aliphatic tail of an amphiphile also affects drug entrapment efficiency. The comparatively greater drug entrapment efficiency of NODNH-18 may be due to its larger lipophilic carbon chain length which increases its lipophilicity and molecular weight (Hao et al., 2002).

#### 3.2.3. In vitro drug release profile

**Figure 4.**
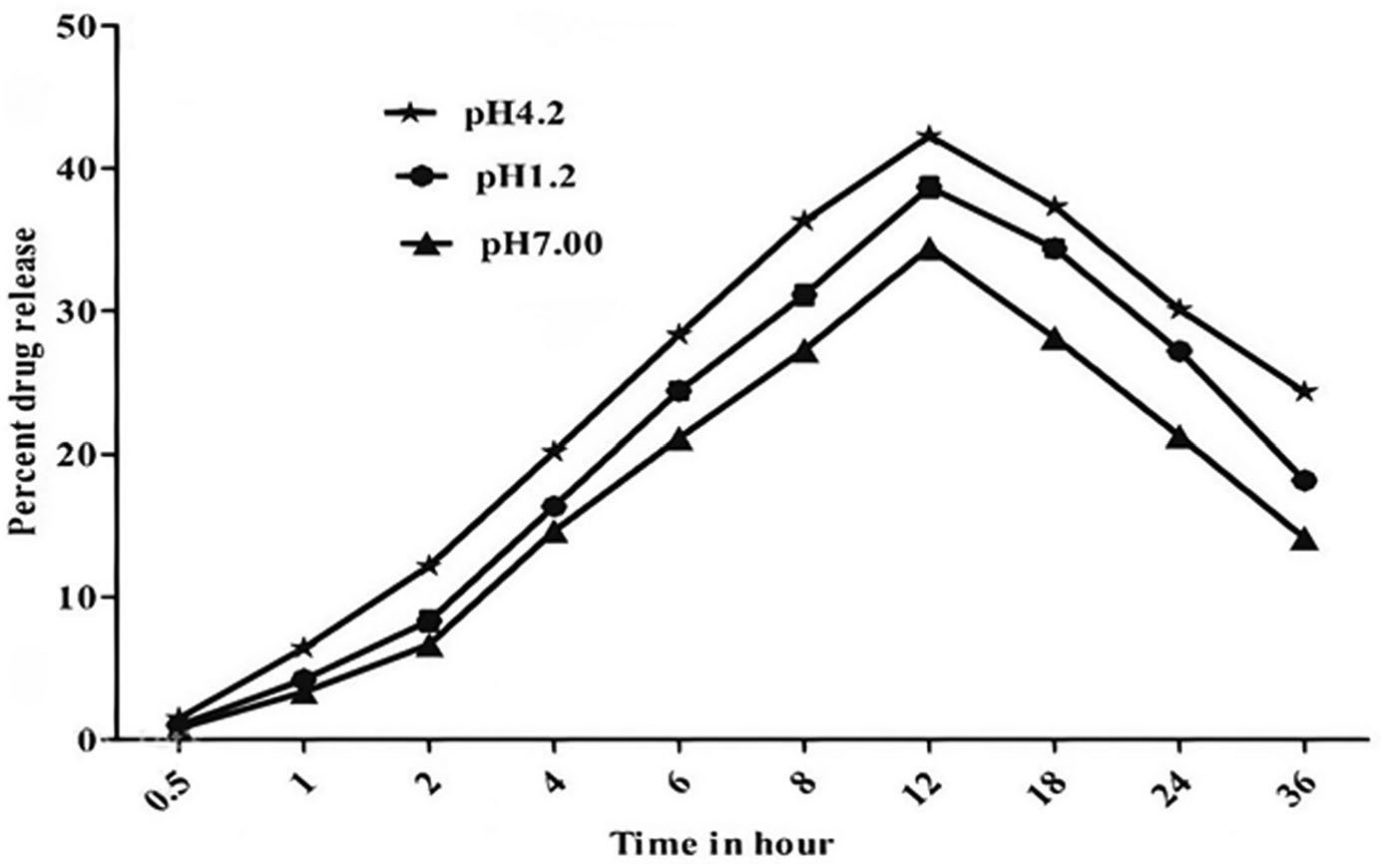
Drug-released behavior of drug-loaded niosomal vesicles of NODNH-18.

**Figure 5.**
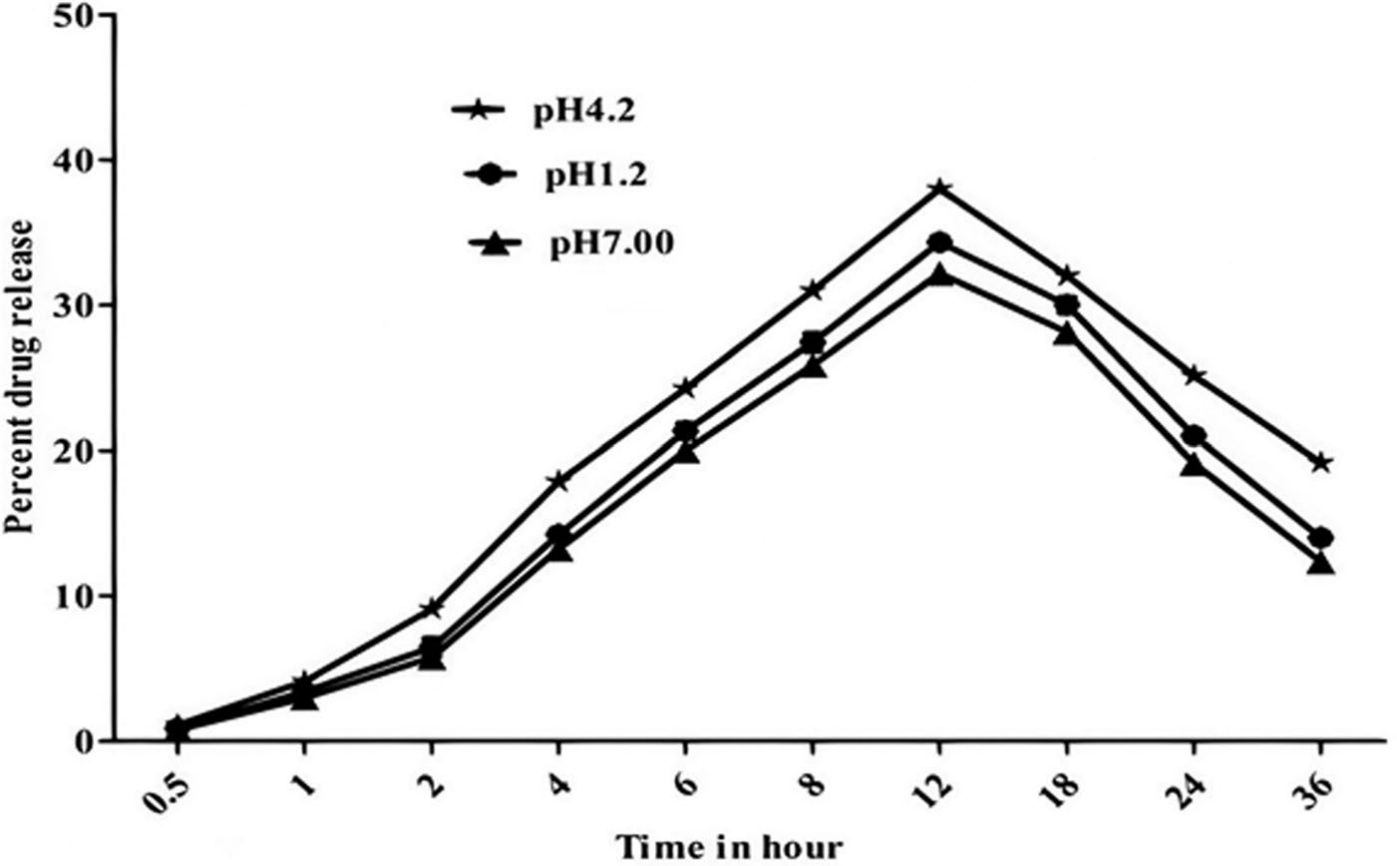
Drug-released behavior of drug-loaded niosomal vesicles of NODNH-16.

The drug release pattern of drug-loaded vesicular formulation of NODNH-16 and NODNH-18 was invested at three different pH (i.e., pH 7.0, 4.2, and 1.2). The drug-loaded vesicular formulation of NODNH-18 showed the slow-release behavior of the drug. It was noted that the highest amount (i.e. 38.67 ± 0.6%) of the drug (amphotericin B) released at pH 1.2 was after 12 h of the study whereas the maximum drug released at pH 4.2 was observed as 42.25 ± 0.5% after 12 h. At pH 7.0, the maximum amount of drug release was found to be 34.41 ± 0.6 at the 11th hour of the study as shown in Figure 4. Similarly, the maximum amount of drug released at three different pH was observed as 34.32 ± 0.4, 38.00 ± 0.7, and 32.18 ± 0.6% at pH 1.2, pH 4.2, and pH 7.00 after 12 h as shown in Figure 5. The slow behavior of drug release from drug-loaded vesicles may be because the carrier bound the drug strongly and released it very slowly (Uchegbu & Vyas, 1998; Jiang et al., 2006). The presence of cholesterol in the vesicular formulation also prevents drug leakage as it works as a cementing agent in the vesicular formulation (Kazi et al., 2010).

#### 3.2.4. FTIR analysis

**Figure 6.**
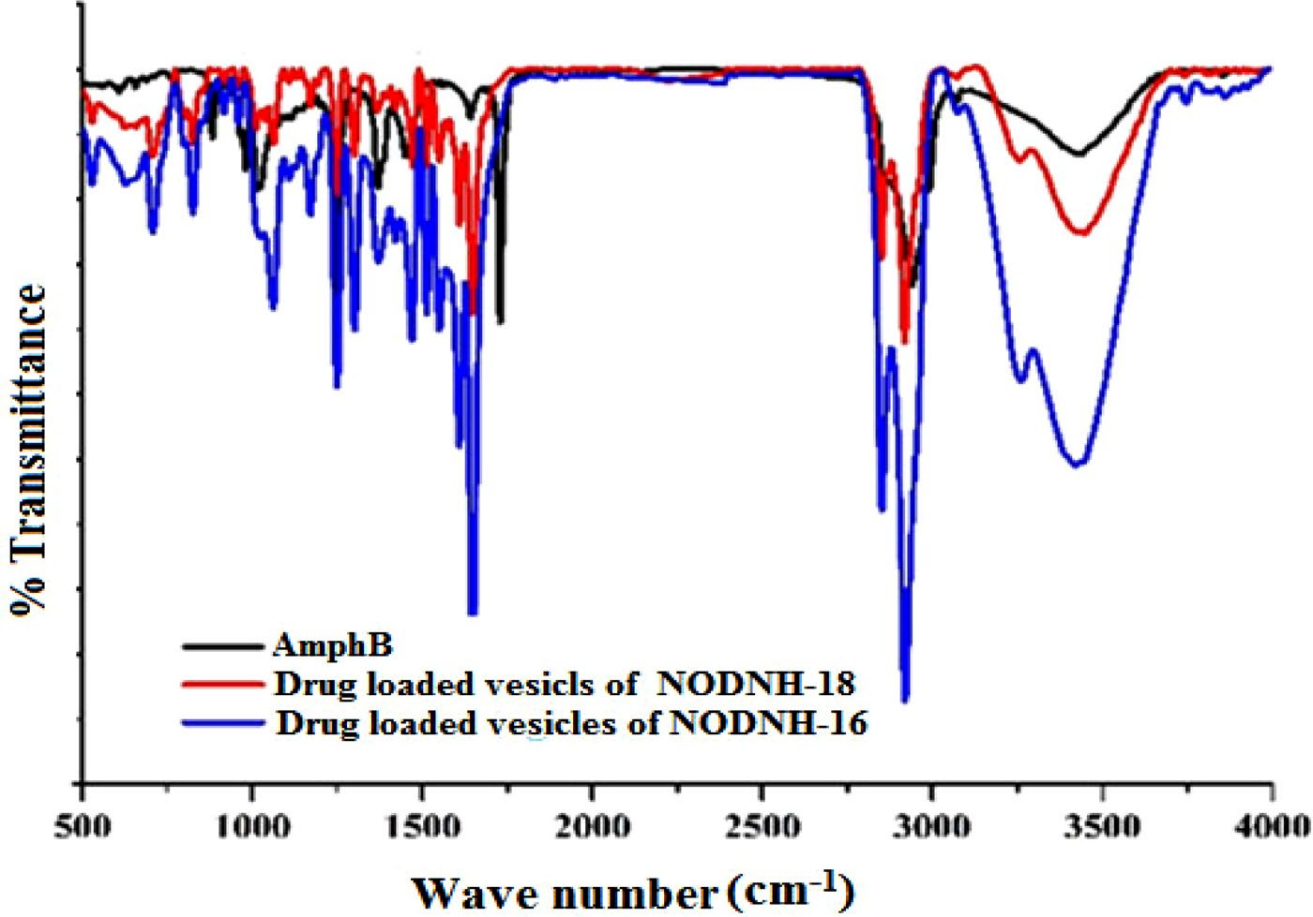
FTIR spectra of drug-loaded niosomal vesicles of NODNH-16 and NODNH-18.

To elevate the possible drug–excipient interactions, FTIR spectra of the drug (amphotericin B), cholesterol, synthesized surfactant carriers, and a dried vesicular formulation containing all ingredients were performed. The FTIR of the drug show their characteristic peak at 3387 cm^−1^ for OH, at 2926 cm^−1^ for NH_2_, at 1712 cm^−1^ for CH_2_, at 1626 cm^−1^ for carbonyl group, at 1550 cm^−1^ for alkene and the bands at 1249.4 cm^−1^ represent C–O stretch in the ester group. The absorption peak at 3416 cm^−1^ and 1669.7 cm^−1^ indicates the characteristic peaks for -OH and CH = CH groups respectively in the cholesterol. The FTIR spectra of the drug carrier (compound) show a peak at 3268 cm^−1^ which is responsible for H-N, at 2918.6 cm^−1^ for CH_3_, at 2850.4 cm^−1^ for CH_2_, 1651 cm^−1^ for carbonyl group of amides, at 1608.0 cm^−1^ for H-N bending vibrations as shown in ***Figure 6***. The absorption band appeared at 3420.9 cm^−1^ in the FTIR spectrum of formulation indicating that the characteristic peak of OH of the pure drug was slightly shifted toward a lower frequency, the peak of the H-N amine group appeared at 3261.0 cm^−1^ which indicated the frequency shifted slightly toward high frequency, and the CH_3_ peak at 2918 cm^−1^, CH_2_ at 2850 cm^−1^, carbonyl group of amide at 1651 cm^−1^, H-N at 1295 cm^−1^ and C-N at 1247 cm^−1^ appeared nearly in the same region at 2920.1 cm^−1^, 2851.6 cm^−1^, 1648.2 cm^−1^, 1300.1 cm^−1^, and 1249.4 cm^−1^ respectively. The main characteristic peaks of the pure drug were present in the formulation spectrum indicating the presence of the drug in an intact form in the formulation. A small change i.e. higher or lower frequency shift was noted which indicated the physical interaction of the drug and carrier (L. Hu et al., 2015).

**Figure 7.**
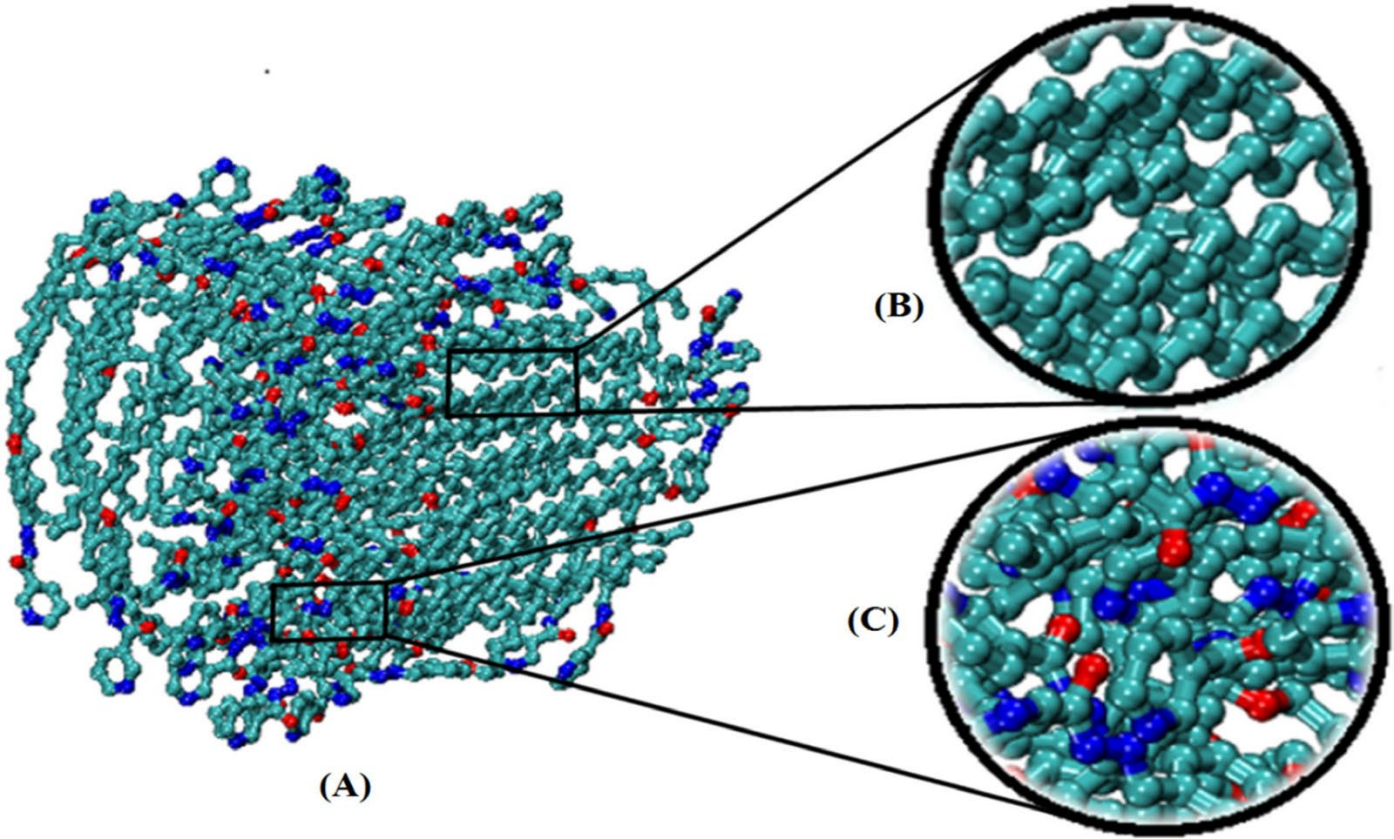
(A) A snapshot of a synthesized surfactant-based vesicle at 30 ns; (B) the center of the vesicle where non-polar tails are mixed up, and (C) the outer surface of the vesicle. The water molecules and hydrogen atoms are omitted for clarity.

#### 3.2.5. Molecular dynamic simulation

Numerous studies and experimental techniques have been performed to elucidate and characterize vesicles’ structures, their dynamic behavior, and thermodynamic properties as they are gaining considerable applications in diverse technological and fundamental fields (Burkhardt et al., 2007; Callari et al., 2017). In recent years, MD simulation studies have ascertained more priceless information contrary to experimental results at atomic levels covering time scales ranging from nanoseconds to microseconds. Various simulation studies have been performed on the self-assembly of amphiphiles to get insights into the exact assembly phenomenon (Smit et al., 1991; Moussa, 2017). Our main interest in performing MD studies was to study the self-assembly process of the synthesized surfactants and to investigate how the vesicle shape depends on molecular size using atomistic MD simulations. MD simulation was used to track trajectories of surfactant atoms that are produced in response to inter and intermolecular forces over a certain period (Moussa, 2017). The shapes of the NODNH-16 and NODNH-18 vesicles were assessed, and snapshots of typical configurations were taken as shown in ***Figure 7***. It is clear that the lipophilic alkyl chains move toward the vesicle center and the hydrophilic head groups orient themselves in such a manner to make an outer covering thereby shielding the lipophilic core from the surrounding water environment. As shown in ***Table 2***, the different snapshots at various time frames were taken, showing how these molecules come close to each other forming spherical vesicles. These MD simulation studies are in great agreement with experimental ones and justify that the synthesized surfactants can assemble to form stable spherical vesicles.

**Table 2.** The size, zeta potential, and polydispersity index (PDI) stability at 4 °C were followed up to 25 days.

Sample	Attribute	Day-1	Day-7	Day-15	Day-25
NODNH-16	PDI	0.28	0.36	0.41	0.38
	Zeta potential (mV)	–19.6 ± 0.9	–17.3 ± 4.59	–23.11 ± 0.83	–15.8 ± 1.52
	Average size (nm)	95.34 ± 2.21	98.41 ± 1.61	107.01 ± 4.51	109.42 ± 3.61
NODNH-18	PDI	0.29	0.32	0.38	0.42
	Zeta potential (mV)	–18.8 ± 0.8	−21.04 ± 0.73	–19.6 ± 1.19	–16.5 ± 2.71
	Average size (nm)	96.13 ± 2.35	102.82 ± 4.71	112.39 ± 1.21	116.92 ± 2.91

**Figure 8.**
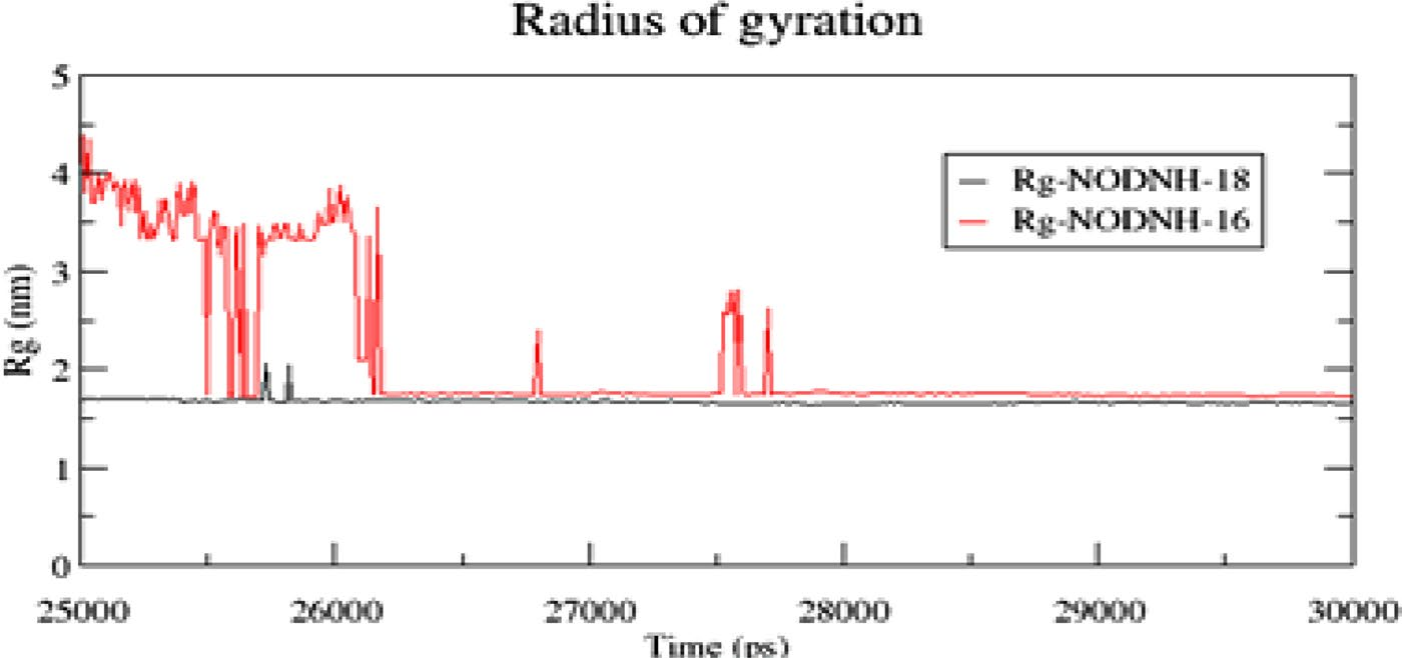
The radius of gyration of the spherical micelles for the last 5 ns of the simulation.

In the case of NODNH-18, all fifty molecules assemble to make a single compact vesicle confirming their small and smooth radius of gyration after 25 ns, while the same happened for NODNH-16 at 27 ns. There is not much difference in the radius of gyration for both the synthesized compounds as shown in ***Figure 8***.

### 3.3. Biocompatibility study

#### 3.3.1. Blood hemolysis assay

**Figure 9.**
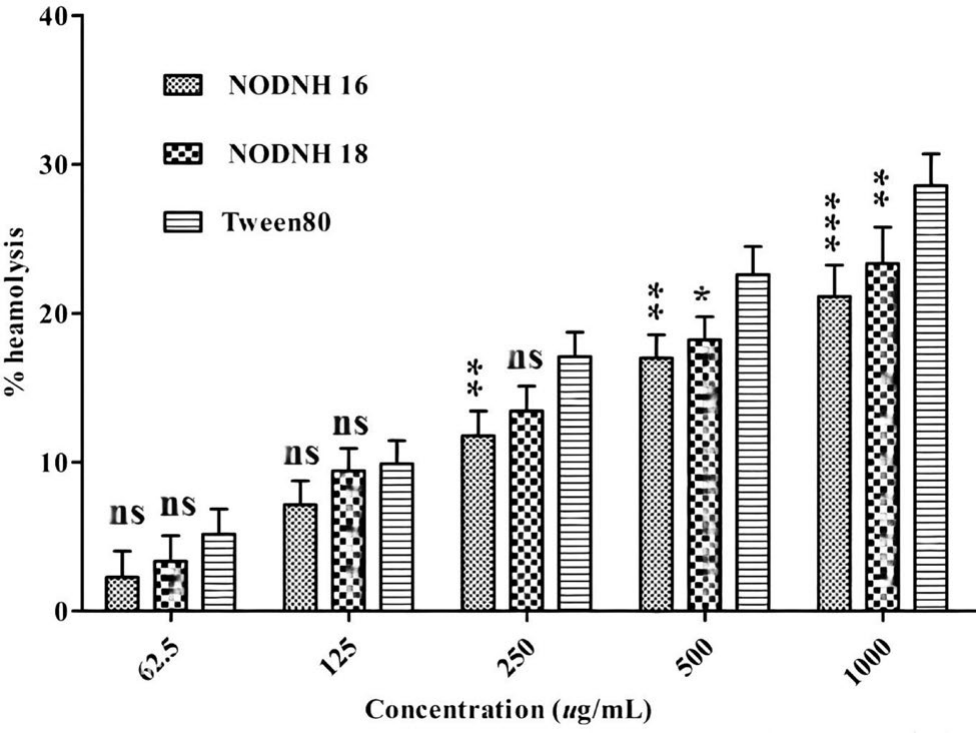
Percent blood hemolysis activity of NODNH-16, NODNH-18, and Tween-80.

The synthesized nicotinic hydrazine-based surfactants were screened for blood hemolysis before use as drug carriers for improving therapeutic efficiency and enhancing the aqueous solubility of low water-soluble drugs. Both synthesized nonionic surfactants showed less hemolysis even at the highest concentration of 1000 µg/mL as compared to Tween®-80 which was used as a reference. The nonionic surfactant NODNH-16 showed 21.13 ± 2.11% hemolysis and NODNH-18 showed 23.32 ± 2.45% whereas the Tween®-80 showed greater hemolysis at 28.57 ± 2.13% at the highest tested concentration of 1000 µg/mL after a 4-h incubation as shown in ***Figure 9***. From these results, the newly synthesized nonionic surfactants are hemocompatible and safe for use in drug delivery. Their hemocompatible character may be due to their nonionic nature as described elsewhere (Ali et al., 2017).

#### 3.3.2. Cell cytotoxicity

**Figure 10.**
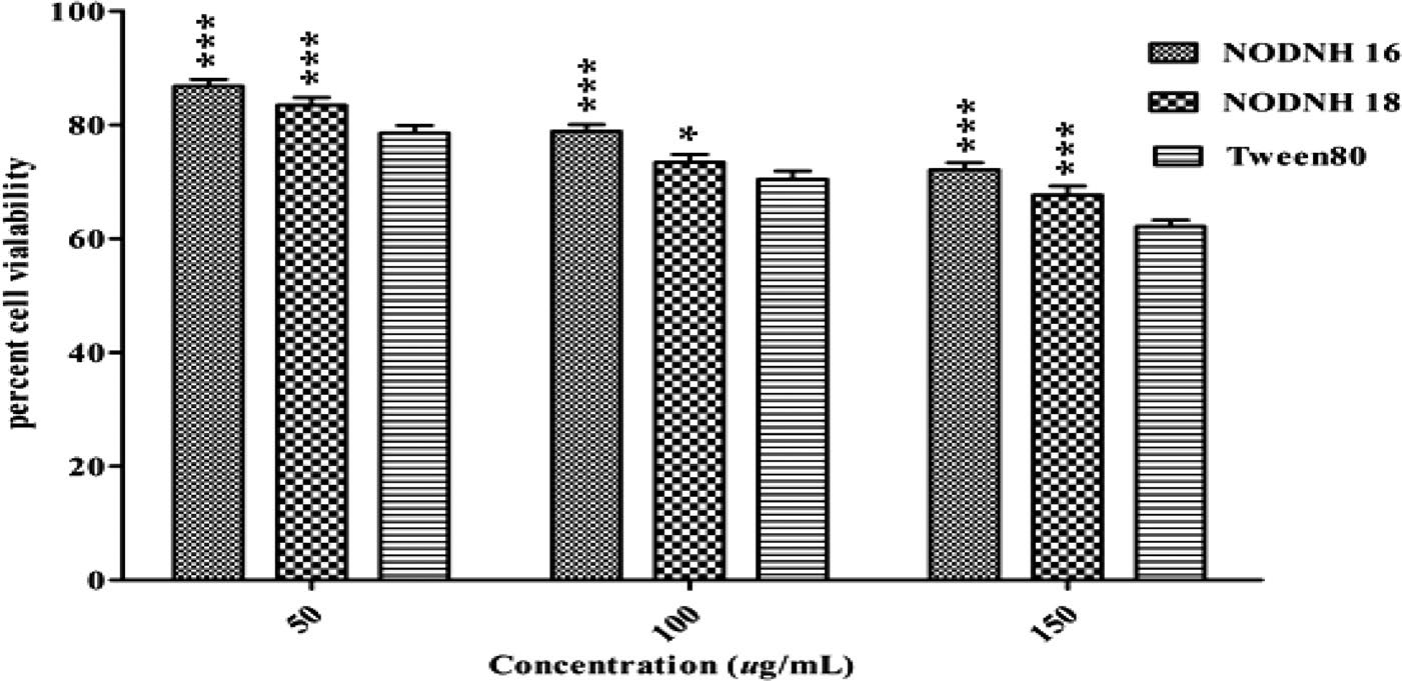
Percent 3T3 cells viability against synthesized surfactants NODH-16, NODH-18, and Tween®-80 at different concentrations.

The newly synthesized nonionic surfactants NODNH-16 and NODNH-18 were screened for a 3T3 cell line toxicity assay. Different concentrations of NODNH-16 and NODNH-18 ranging from 50 to 150 µg/mL were subjected to a 3T3 cell line to check their cytotoxicity. The newly synthesized nonionic surfactants NODNH-16 and NODNH-18 showed higher cell vial ability even at the highest tested concentration of 150 µg/mL and the viability of the cells was observed as 72.12 ± 1.24% and 67.67 ± 1.56% respectively whereas the reference standard Tween®-80 showed 62.13 ± 1.12% 3T3 cells viability at this concentration as shown in ***Figure 10***. The lower cytotoxicity of both newly synthesized nonionic surfactants may be due to their appropriate chain length and nonionic nature as described in the literature (Gershanik & Benita, 2000). Previous reports indicate that amphotericin B exhibits cytotoxicity in the fibroblast lineages (Harmsen et al., 2011; Noor et al., 2022). In our study, the niosomes NODNH-16 and NODNH-18 mitigate the fibroblast toxicity as compared to previous reports on the toxic levels of conventional amphotericin B.

### 3.4. Antifungal activities

***Table 3*** exhibits the fungicidal activities of standard drugs i.e. amphotericin B, fluconazole, nystatin, and carrier niosomes (NODNH-16 and NODNH-18) containing amphotericin B NODNH-16 and NODNH-18 carrier niosomes augments the fungicidal activity of amphotericin B via increasing the zones of growth inhibition (ZOI) against the human pathogenic yeast species as well as multi-cellular fungal species except the *F. oxysporum* and *P. variotii* (Table 3). The highest ZOI indicated by NODNH-16 against the *S. cerevisiae* 26 (±1.09) followed by *C. albicans* ATCC 10231 21 (±0.87), *C. galeberata* ATCC 20001 19 (±0.92), *M. guilliermondi* 16.7 (±0.51), and *T.* longibrachi 17 (±1.2). However, NODNH-18 revealed an increase in ZOI against the *S. cerevisiae* 30 (±0.81) followed by *C. galeberata* ATCC 20001 22(±0.31), *T.* longibrachi 20 (±1.1), *C. albicans* ATCC 10231 18(±0.2), *A. terreus* 18.1(±0.79), and *P. lilacinum* 18 (±0.08), respectively (Table 3). Furthermore, inhibitory zones produced by the NODNH-16 and NODNH-18 were close to their mean averages (estimated in the triplicate experiments), and a decrease in standard deviation was observed as compared to free amphotericin B (Table 3). It is noteworthy, that the concentration of free drug used to assess fungicidal activities at pH 5.6 for 24–48 h against the human pathogenic and environmental fungi was 15 µg as compared to the entrapped amphotericin B in vesicle NODNH-16 and NODNH-18, i.e. 60.89% (9.13 µg) and 68.63% (10.29 µg) respectively. Although the entrapped amount of amphotericin B was 60 to 68% and the drug release behavior at pH 4.2 (close to the pH of the fungicidal assay) was 30 to 42.25 ± 0.5% after 12 h, the vesicle likely enhance the drug activity at low dosages primarily due to increasing the availability for the growth inhibition. Amphotericin B is a hydrophobic drug as compared to fluconazole and nystatin. The solubility of amphotericin B might compromise in the aqueous cellular environment, however larger lipophilic carbon chains in NODNH cause the internalization of amphotericin B via hydrophobic interaction and forming niosomes (Noor et al., 2022). Reducing the surface area of the drug in the physiological environment may increase the transportation of fungicides inside the cell and improve the function of the drug. Whereas, the NODNH off load the amphotericin B causing an increase in cell permeability via binding with the ergosterol (Alqahtani et al., 2021).

**Table 3.** Snapshots of surfactant molecular assemblies and their spontaneous aggregation into spherical vesicles at various time points.

Time	NODNH-16	NODNH-18
0 ns	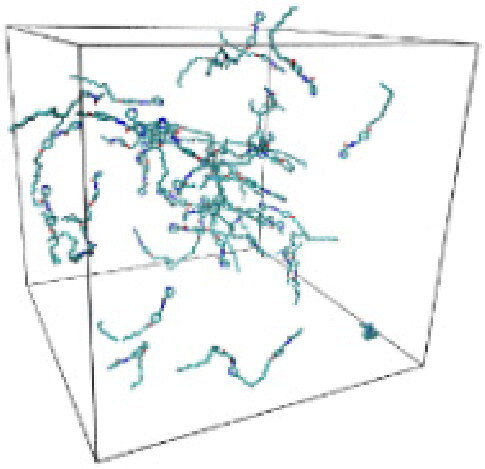	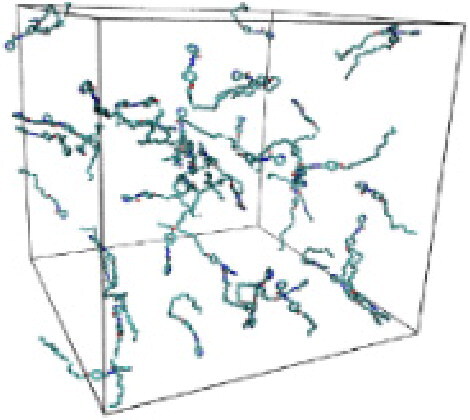
5 ns	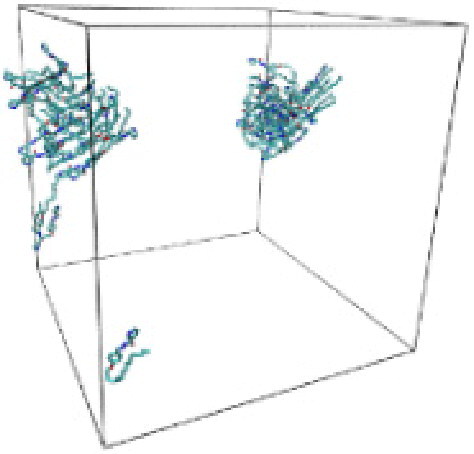	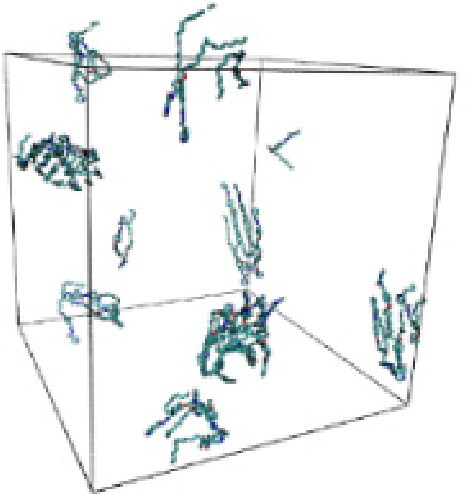
10 ns	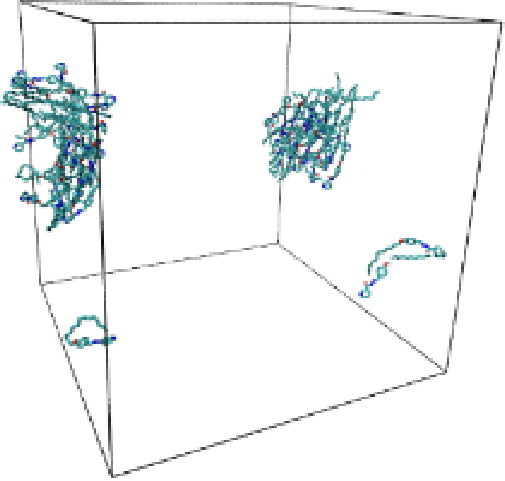	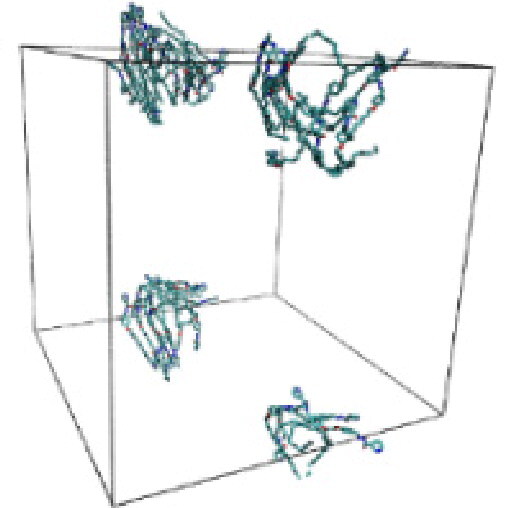
15 ns	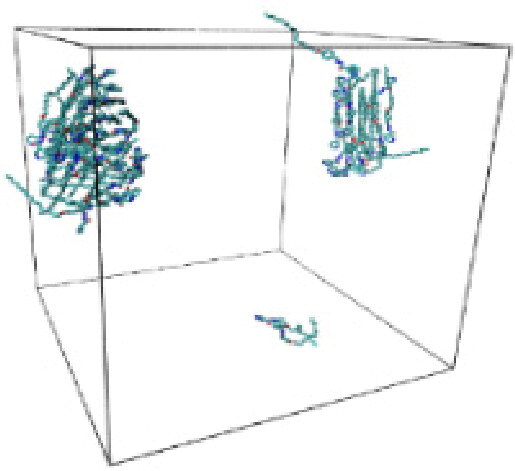	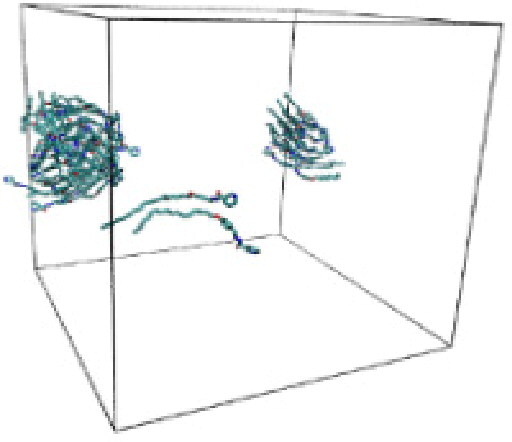
20 ns	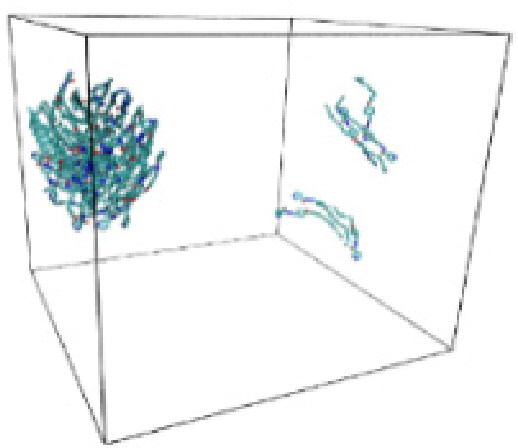	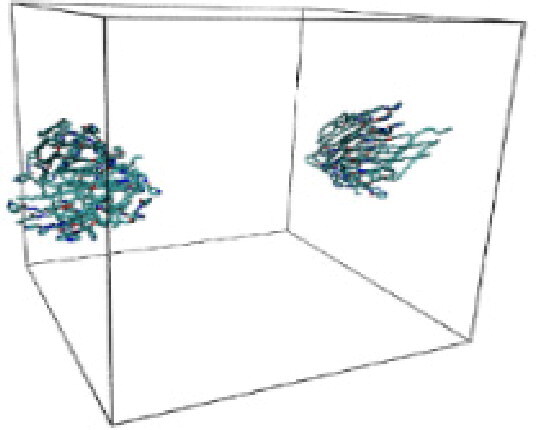
25 ns	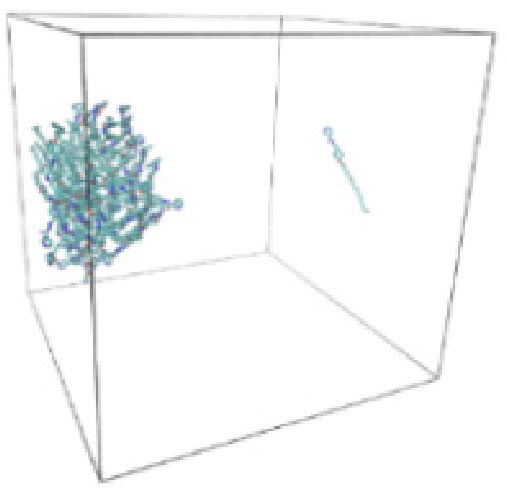	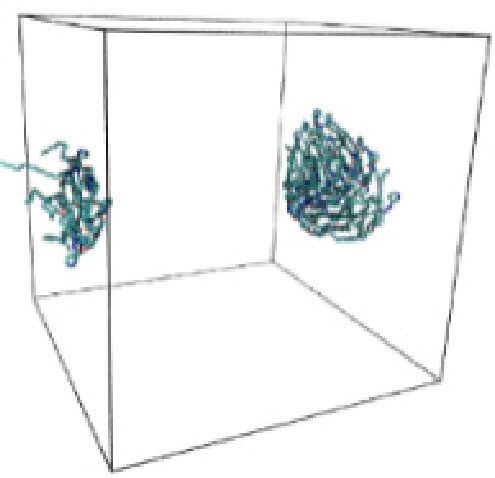
30 ns	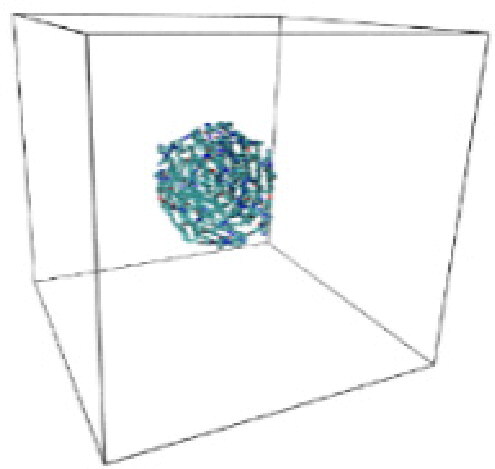	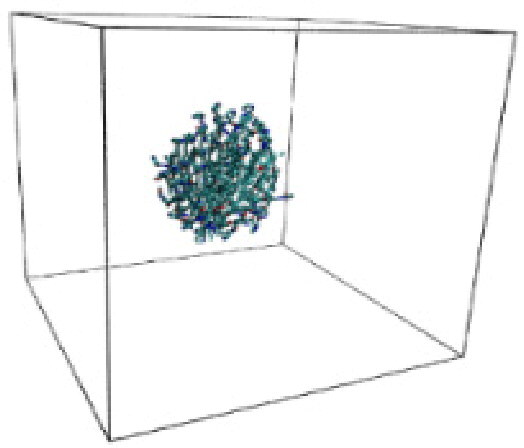

Water molecules and hydrogen atoms are omitted for clarity.

AFI of the carrier vehicle NODNH-16 exhibits an enhanced efficacy of amphotericin B against human pathogens, i.e. *C. albicans* ATCC 10231, *C. galeberata* ATCC 20001, and *M. guilliermondi* (***Table 4***). In addition, NODNH-18 reveals a greater fungicidal index against the fluconazole*-*resistant *C. galeberata* ATCC 20001 as compared to NODNH-16. Furthermore, the AFI for the NODNH-16 and NODNH-18 also compliments the fungicidal activities against multi-cellular fungal species, presented in Table 5. However, 18 carbon-containing NODNH-18 niosomal vesicle exhibits more effective delivery of amphotericin B which leads to an increase in AFI for *T. longibrachiatum, P. lilacinum,* and *A. Terreus* (Table 5).

**Table 4.** Zones of inhibition of standard fungicidal drugs and carrier vesicles NODNH-16, NODNH-18 loaded with amphotericin B.

Organisms	Zones of Inhibition in millimeters (mm)				
	Amphotericin B (mm)	Fluconazole (mm)	Nystatin (mm)	NODNH-16 (mm)	NODNH-18 (mm)
*C. albicans* ATCC 10231	10 ± 2.1	15.3 ± 1.1	14 ± 1.90	21 ± 0.87	18 ± 0.20
*C. galeberata* ATCC 20001	14 ± 1.8	2 ± 1.30	12 ± 2.10	19 ± 0.92	22 ± 0.31
*T.* longibrachiatum	18 ± 2.5	11 ± 2.31	14 ± 4.03	17 ± 1.20	20 ± 1.10
*P. lilacinum*	12 ± 2.3	13.7 ± 2.01	11 ± 1.67	12.8 ± 0.19	0.08
*A. Terreus*	16.3 ± 3.3	13 ± 1.90	09 ± 2.07	15 ± 0.91	18 ± 0.79
*P. variotii*	12 ± 4.10	07 ± 3.89	11 ± 2.71	14 ± 1.01	11 ± 0.91
*F. oxysporum*	–	14 ± 1.90	19 ± 1.97	–	–
*M. guilliermondi*	09 ± 1.90	15 ± 1.02	13.4 ± 1.70	16.7 ± 0.51	14.9 ± 0.23
*S. cerevisiae*	21.3 ± 3.60	23 ± 2.70	28 ± 1.92	26 ± 1.09	30 ± 0.81

ZOI (mm) = Zones of inhibition calculated in millimeters, - = Not observed.

**Table 5.** Anti-fungal index (AFI) of the carrier vehicles loaded with amphotericin B.

Organisms	NODNH-16 AFI	NODNH-18 AFI
*C. albicans* ATCC 10231	2.01	1.8
*C. galeberata* ATCC 20001	1.35	1.57
*T. longibrachiatum*	0.94	1.11
*P. lilacinum*	1.06	1.5
*A. terreus*	0.92	1.11
*P. variotii*	1.16	0.91
*F. oxysporum*	–	–
*M. guilliermondi*	1.85	1.65
*S. cerevisiae*	1.22	1.408

AFI = Zone of inhibition of amphotericin B loaded liposomal vesicles/zone of inhibition of amphotericin B, - = Not observed, AFI> 1 = indicate the effectiveness of the drug.

## 4. Conclusion

The nonionic surfactants have got the great attention of pharmaceutical scientists in recent years due to their greater biocompatible nature. Nonionic surfactants form closed bilayer vesicles or micellar structures upon their contact with an aqueous environment. Due to their remarkable capacity and properties such as biocompatibility, cost-effectiveness, and stability, these vesicles are regarded as excellent carrier for the delivery of drugs. Two newly synthesized nicotinic hydrazine-based nonionic surfactants have been screened out here for CMC, biocompatibility, and their potential for drug encapsulation and release was evaluated. Both the synthesized nonionic surfactants NODNH-16 and NODNH-18 have a great ability to entrap the greater amount of model hydrophobic drug amphotericin B. Drug-loaded vesicles of the synthesized surfactants were of small size with less polydispersity index value. From the results, it can be concluded that both newly synthesized nonionic surfactants are promising candidates to be used for vesicular drug delivery of less water-soluble drugs like Amphotericin B and showed a significant potential for fungicidal activity.

## Data Availability

NA

## Sample Availability

NA
